# Case study documenting the diagnosis of idiopathic CD4+ Lymphocytopenia in a patient with atypical fungal infection (disseminated blastomycosis) by FNA of adrenal mass

**DOI:** 10.4103/1742-6413.67106

**Published:** 2010-08-05

**Authors:** Richard H Siderits, Osman Ouattara, Alan Marcus, Hong Guang Gao, Hong Bing Deng, Janusz Godyn

**Affiliations:** Robert Wood Johnson University Hospital Hamilton, New Brunswick, USA; 1 Robert Wood Johnson Hamilton Cytopathology Service, Hamilton, New Brunswick, USA; 2 Robert Wood Johnson University Medical School, New Brunswick, USA

**Keywords:** Blastomycosis, idiopathic, lymphocytopenia

## Abstract

Idiopathic CD4+ lymphocytopenia, described in 1992 by the Centers for Disease Control, is characterized by persistent CD4+ lymphocytopenia (less than 300 cells per micro-liter) in nonimmunosuppressed, HIV negative individuals, who present with atypical infections. This rare though likely undiagnosed entity is associated with chronic disseminated forms of either fungal or bacterial infections in otherwise healthy adults. We report a case of a 59-year-old male with ring-enhancing brain lesions, bilateral adrenal masses, lung and vocal cord nodules, where the diagnosis of exclusion was metastatic malignancy. Fine needle aspiration (FNA) of the adrenal mass and a subsequent vocal cord biopsy confirmed chronic widely disseminated blastomycosis. Flow cytometric evaluation of peripheral blood documented persistent selective CD4+ lymphocytopenia with T8 (suppressor) T-Lymphocyte count within normal range. We believe that idiopathic CD4+ lymphocytopenia is an important etiologic factor to be considered for patients who present with mass lesions and are diagnosed by FNA with atypical fungal infections. We relate the diagnostic criteria for idiopathic CD4+ lymphocytopenia and the importance of providing on-site triage for FNA samples for fungal studies and correlation for flow cytometry.

## INTRODUCTION

In 1992, the CDC identified a group of patients who were human immunodeficiency virus (HIV) negative and nonimmunosuppressed, however, presented with selective and sustained CD4+ lymphocytopenia and atypical fungal or bacterial infections. They defined the process as Idiopathic CD4+ Lymphocytopenia (ICL) and set specific criteria for diagnosis. The diagnosis rested upon identifying atypical infections, in otherwise healthy individuals, who had persistent CD4+ lymphocytopenia (less than 300 cells per micro-liter) and were non-immunosuppressed and were HIV negative. This entity has remained relatively rare although most likely underdiagnosed.

## CASE REPORT

**Figure 1:**
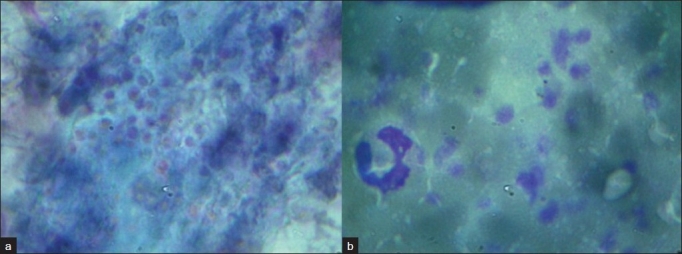
a) Papanicolaou stained smears from diagnostic adrenal gland FNA which show a punctate appearance of fungal yeast within a background of partially obscuring blood and necrotic material (Oil), b) Modified Wright Giemsa stained smears from diagnostic adrenal gland FNA showing a punctate appearance of fungal yeast within a background of partially obscuring blood and necrotic material (Oil)

We report a case of a 59 -year-old caucasian male with no significant past medical history who presented to the emergency department with a six-week history of weakness, loss of appetite and “hoarseness” of voice. His employment history showed that he worked in air conditioning repair and was often in basements and attics. Laboratory values upon admission were noncontributory with white blood cell counts and hemoglobin level, in normal ranges. Chest X-ray in the emergency department showed an early nonspecific nodular-interstitial infiltrate. Computerized tomography (CT) obtained upon admission revealed a left adrenal mass and multiple ring enhancing lesions in brain. Based on these findings the differential diagnostic impression was that of metastatic carcinoma. An adrenal gland FNA was performed. On-site evaluation of modified Wright-Giemsa stained smears for specimen adequacy revealed necrotic granular material with a speckled vacuolar appearance suggestive of fungal yeast. Several passes were obtained, however, it was not possible to confirm malignancy [Figure 1a and b]. The possibility of an infective process was considered and samples for microbiologic evaluation were obtained.

**Figure 2:**
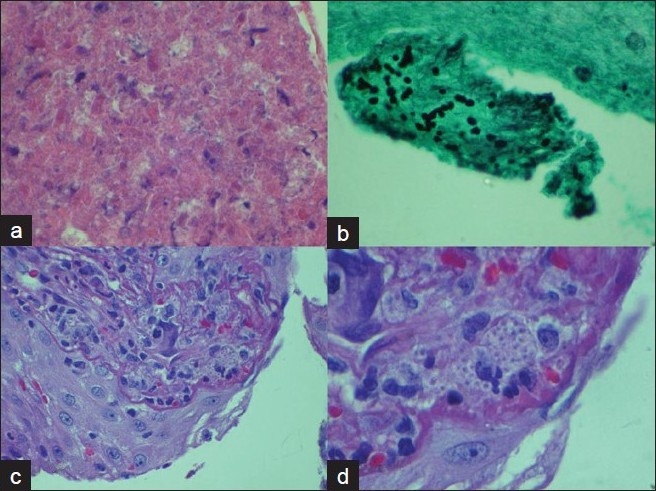
a) (Top Left): FNA adrenal mass with necrotic eosinophilic granular material; Hematoxylin and Eosin stain of cell block at 20× Magnification b) (Top Right): Scattered broad based budding fungal yeast; Gomori Methenamine Silver stain under Oil. c) (Bottom Left): Vocal cord Biopsy Hematoxylin and Eosin Stain at 20× magnification with squamous atypia. d) (Bottom Right): Vocal cord Biopsy Hematoxylin and Eosin Stain at 40× magnification with subepithelial fungal yeast).

The cell block preparation from the FNA procedure revealed rare broad based budding yeast [Figure 2a and b]. Bronchoscopy performed to evaluate the nature of the interstitial infiltrate documented multiple small ulcerated areas on the vocal cords. These ulcerated areas were biopsied and showed marked squamous atypia with subtending aggregates of broad based budding yeast. Subsequent evaluation of Papanicolaou (PAP) stained smears, Gomori Methenamine-Silver Nitrate Stained (GMS) and Hematoxylin-Eosin (H and E) stained cell block material showed clusters of broad based budding yeast, consistent with Blastomycosis. These findings, in conjunction with the adrenal FNA suggested a widely disseminated (systemic) fungal infection [Figure 2c and d]. Urine Histoplasma Antigen screen, obtained concurrently, was reported as Positive. In light of these findings in an otherwise healthy individual an HIV test was performed and was negative (repeat HIV testing, by serology and Western blot, remained negative).

The possibility of ICL was entertained and Flow cytometry of peripheral blood, performed at our request, documented selective CD4+ Lymphocytopenia (155 cell/microliter) with T8 (suppressor) population in normal range. This supported the diagnosis of ICL with associated atypical fungal infection. Fungal culture confirmed Blastomyces *dermatitidis*. The final diagnosis was that of Idiopathic CD4+ Lymphocytopenia with disseminated Blastomycosis involving adrenal gland, vocal cord and probable lung and brain, in an HIV negative non-immunosuppressed patient.

Parenthetically, the T4 lymphocytopenia has remained decreased (<300) for more than two years. Treatment included Intravenous amphotericin B followed by Amphoteracin-B Lipid complex. Patient was placed on oral itraconazole, 200 mg per day for 18 months as per IDSA guidelines for disseminated blastomycosis, with monitoring for blood levels once per month. An additional lesion had been identified in the right hip with FNA documented Blastomycosis. The lung, brain and vocal cord lesions have remained essentially unchanged.

## CONCLUSIONS

Idiopathic CD4+ Lymphocytopenia is considered rare and is rarely considered within the differential diagnosis for Fine Needle Aspirations of otherwise healthy individuals with atypical fungal infections who present with mass lesions. The CDC has defined this entity as sustained CD4+ Lymphocytopenia (less than 300 CD4+ lymphocytes per micro liter) with persistent T4 lymphocytopenia following treatment (or resolution of infection) without immunosuppression in HIV negative individuals. ICL is now considered to an underdiagnosed etiology for atypical fungal infections.[1] It is most likely not caused by a response to infectious etiologies (which are characterized by transient suppression of T-Cell subsets).[2] The severity of ICL varies from asymptomatic to life threatening.[2] Idiopathic CD4+ lymphocytopenia is associated with several clinical entities including: Burkitt’s lymphoma, progressive multifocal leukoencephalopathy, disseminated cryptococcosis, relapses of leishmaniasis after treatment, *Mycobacterium avium-intracellulare*, cryptococcal meningitis.[3] One study found that the most common infections associated with ICL was either cryptococcosis or nontubercular mycobacterial infections.[4]

The patient described in this paper presented with mass lesions diagnosed as a chronic disseminated form of Blastomycosis having involved adrenal, brain, vocal cord, lung and bone. Incidentally, pulmonary infection in patients with suppressed or low T4 counts can result in adult respiratory distress syndrome, septic shock and death with a mortality rate (in immunocompromised patients) greater than 30%.[5,6]

It is important to consider, in passing, that atypical fungal infections in patients with ICL may show misleading information such as: 1) malignancy as the diagnosis of exclusion atypical fungal infections presenting with multi organ “mass” lesions; 2) 80% cross reactivity with both Histoplasma and Cryptococcus antigens in screening tests; and 3) up to 60% false negative serology in patients with local atypical fungal infection (i.e., blastomycosis).

Other organs commonly involved by blastomycosis include the skin and bone, urinary tract.[7–12] Involvement of the Adrenal gland in disseminated blastomycosis, may rarely lead to Addisonian crisis.[13,14] Diagnostic methods include wet preparation, cytologic, histologic and serologic evaluation and the gold standard, fungal culture.[15,16]

In the immunocompromised or immunosuppressed patient, fungal infections can be severe and prompt diagnosis with appropriate treatment is essential.[17] The treatment for blastomycosis in immunodeficient patients is Amphotericin B.[18] There have recently been efforts to create a vaccine against *Blastomyces dermititidis*.[19] It has been shown that in immunocompetent patients CD4 T helper cells are a major component in the immune response to *Blastomyces dermititidis*. However, in the absence of CD4 lymphocytes, memory CD8 lymphocytes via the Class- 1 major histocompatiblity complex can recognize the *B. dermititidis* antigen and thereby initiate may immune response. This suggests that *B. dermititidis* vaccines may in fact be beneficial to susceptible patients whom lack CD4 T lymphocytes.[20]

Nevertheless, prophylaxis remains the cornerstone of care for patients diagnosed with ICL.[17] New approaches to treatment have also included work with Interleukin- 2 (IL- 2 ) to increase the CD4+ lymphocyte count.[21,22]

In summary, this paper highlights the importance of considering ICL as a diagnostic entity (especially when on-site FNA evaluation is suggestive of an atypical fungal infection in non-immunosuppressed HIV-negative patients) and the role for on-site specimen adequacy evaluation allowing timely triage of samples for special studies (e.g., flow cytometry).

## COMPETING INTEREST STATEMENT BY ALL AUTHORS:

No competing interest to declare by any of the authors.

## AUTHORSHIP STATEMENT BY ALL AUTHORS:

Each author acknowledges that this final version was read and approved. All authors of this article declare that we qualify for authorship as defined by ICMJE http://www.icmje.org/#author. Each author has participated sufficiently in the work and take public responsibility for appropriate portions of the content of this article.

## ETHICS STATEMENT BY ALL AUTHORS:

As this is case report without identifiers, our institution does not require approval from Institutional Review Board (IRB) (or its equivalent)

## EDITORIAL / PEER-REVIEW STATEMENT:

To ensure integrity and highest quality of CytoJournal publications, the review process of this manuscript was conducted under a double blind model(authors are blinded for reviewers and reviewers are blinded for authors)through automatic online system.
